# Impact of two different intramedullary nail fixations performed through the knee joint on knee osteoarthritis: A retrospective radiological analysis

**DOI:** 10.1097/MD.0000000000043896

**Published:** 2025-08-22

**Authors:** Ahmet Senel, Baris Acar, Ahmet Sinan Kalyenci, Engin Carkci, Sinan Erdogan, Erhan Sukur, Baris Polat

**Affiliations:** a Department of Orthopaedics and Traumatology, Istanbul Training and Research Hospital, Istanbul, Turkey; b Deparment of Operating Room Services, Biruni University, Vocational School, Istanbul, Turkey; c Orthopaedics and Traumatology Department, Girne American University, Kyrenia, Turkish Republic of Northern Cyprus.

**Keywords:** fracture, knee, nail, osteoarthritis, retrograde femur, suprapatellar tibia

## Abstract

Although retrograde femoral (RGF) and suprapatellar approach tibial (SPT) nails performed through the knee joint have shown successful outcomes, their effects on knee osteoarthritis remain unclear. This study aimed to evaluate the radiological effects of RGF and SPT nailing on knee osteoarthritis progression. Between January 2014 and February 2024, 35 patients who underwent RGF (Group 1) or SPT (Group 2) nailing met the study criteria. We retrospectively reviewed digital archives and radiological images and recorded demographic data, trauma type, comorbidities, complications, fracture healing time, and follow-up period. In the radiological evaluation, fracture types were classified. Additionally, preoperative and final postoperative anteroposterior radiographs of the knee joint were used to assess the tibiofemoral joint (TFJ), while lateral radiographs were used to evaluate the patellofemoral joint (PFJ). Both joints were categorized according to the Kellgren–Lawrence osteoarthritis (KLOA) grading system. Changes in KLOA grade between the preoperative period and final follow-up were analyzed statistically. The mean age of the patients was 39.0 ± 13.7 years (range: 19–65 years), with 32 (91.4%) male and 3 (8.6%) female patients. RGF nailing (group 1) was performed in 21 patients (60.0%) and SPT nailing (group 2) in 14 patients (40.0%). The median fracture healing time was 5 months in group 1 and 4.5 months in group 2. KLOA grading indicated a statistically significant change in PFJ osteoarthritis in Group 1 (*P* = .023), but no significant TFJ change (*P* = .059). Group 2 showed no significant KLOA changes in the PFJ and TFJ (*P* = .317 and *P* = .083, respectively). Based on the results of our study, RGF nailing appears to increase the risk of osteoarthritis in the PFJ, whereas SPT nailing does not significantly affect the risk of osteoarthritis in either the PFJ or TFJ. Further comprehensive studies with larger patient cohorts are needed to confirm these findings.

## 1. Introduction

Tibial and femoral fractures are prevalent orthopedic injuries that are frequently caused by high-energy trauma and often result in considerable functional impairment. Intramedullary nailing has become the gold treatment option for managing these fractures owing to its minimally invasive approach and capacity to provide stable fixation.^[1,2]^

Suprapatellar approach tibial (SPT) nailing is a technique increasingly favored for the treatment of tibial shaft fractures owing to its several distinct advantages.^[3]^ Unlike the traditional infrapatellar (IP) approach, the suprapatellar method involves the insertion of the nail through an entry point above the patella within the patellofemoral joint (PFJ). This approach allows for better alignment of the tibial axis, particularly in fractures with significant displacement or deformity, because the knee remains in a semi-extended position during the procedure. This positioning enhances fracture visualization and simplifies nail insertion along the natural curvature of the tibia, reducing the risk of malalignment.^[4,5]^ However, the proximity of the nail to the PFJ raises concerns about the increased risk of patellar chondromalacia, characterized by cartilage softening and degeneration under the patella.^[6]^ Similarly, retrograde femoral (RGF) nailing is the preferred technique, particularly for distal femoral fractures, as it offers easy access to the fracture site.^[7]^ However, the passage of the nail through the knee joint may create biomechanical stress on the PFJ, potentially leading to patellar chondromalacia.^[8]^

This study aims to evaluate the early-mid-term radiological outcomes of patients treated with SPT and RGF nailing, with a specific focus on the incidence of PFJ osteoarthritis. We hypothesize that transarticular nail designs do not cause PFJ and tibiofemoral joint (TFJ) osteoarthritis.

## 2. Patients and methods

This retrospective study was approved by Istanbul Training and Research Hospital Ethics Committee (date: 06/09/2024; no. 71). The inclusion criteria were as follows: patients aged 18 to 65 years, those who underwent SPT intramedullary nailing for tibial diaphyseal fractures or RGF intramedullary nailing for femoral diaphyseal fractures, and patients with available routine postoperative follow-up and radiological imaging. The exclusion criteria were the presence of an intraarticular fracture in the ipsilateral knee, a history of previous knee surgery, prior ipsilateral osteomyelitis or septic arthritis in the knee joint, a history of inflammatory joint disease and, corticosteroid use for any reason after surgery, a follow-up period of <6 months, leg length discrepancy observed in the 6th postoperative month after fracture healing, implants extending into the joint (even by a few millimeters), as seen on postoperative radiographs, and the absence of written informed consent. Between January 2014 and February 2024, 47 patients who underwent SPT and RGF nailing for tibial and femoral diaphyseal fractures in our clinic were reviewed from the hospital records and radiology archives. As a result, 35 patients met the criteria. Patients were divided into 2 groups: Group 1, those who underwent RGF nailing and Group 2, those who underwent SPT nailing.

**Figure 1.**
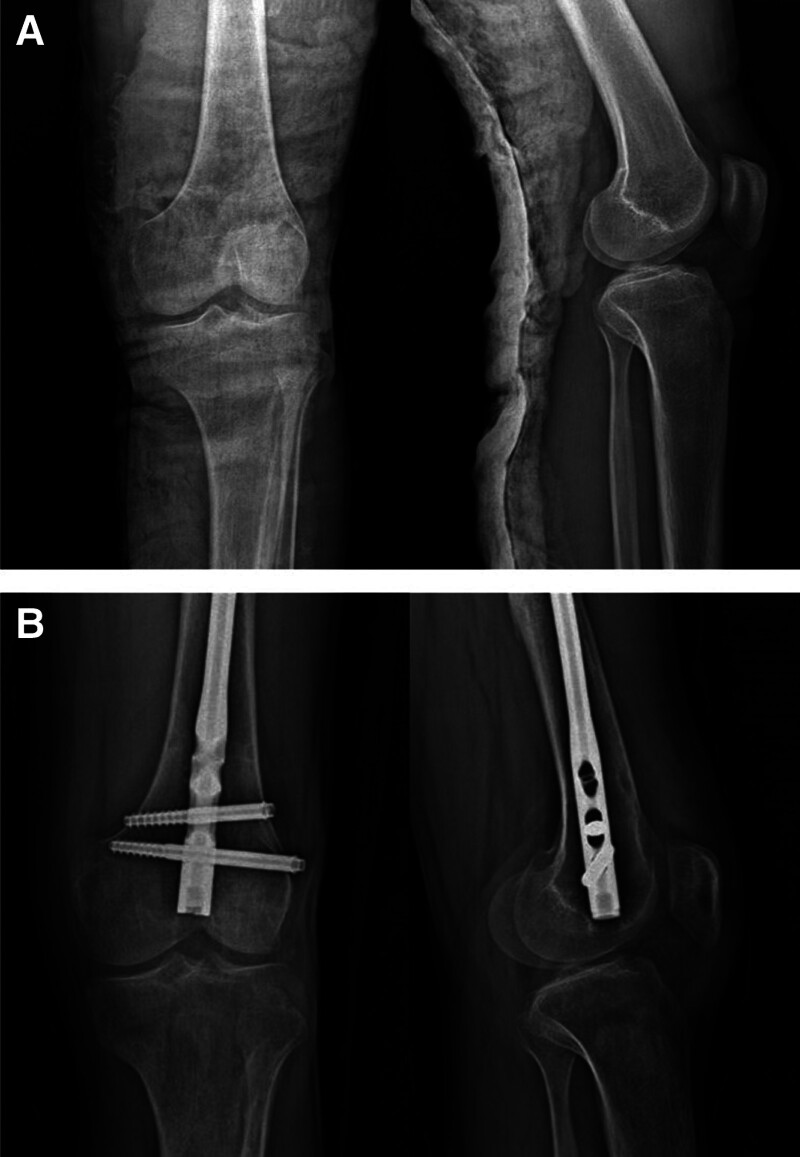
Preoperative (A) and postoperative final follow-up (B) anteroposterior-lateral radiological knee views of a patient who underwent intramedullary nailing due to femoral diaphyseal fracture.

Age, sex, side, type of trauma, comorbidities, accompanying injuries, complications, associated injuries, fracture healing time, and follow-up period were also recorded. In the radiological evaluation, fracture types were classified according to the Arbeitsgemeinschaft für Osteosynthesefragen fracture classification.^[9]^ Additionally, preoperative and final postoperative anteroposterior knee radiographs were used to assess the TFJ, while lateral knee radiographs were used to evaluate the PFJ (Fig. 1). Both joints were categorized according to the Kellgren–Lawrence osteoarthritis (KLOA) grading system.^[10]^ Radiological evaluation and categorization of the patients were performed by 2 authors who were blinded to the demographic and group information. In cases of disagreement, radiological assessments were repeated until a consensus was reached.

### 2.1. Surgical procedure

#### 2.1.1. RGF nailing

**Figure 2.**
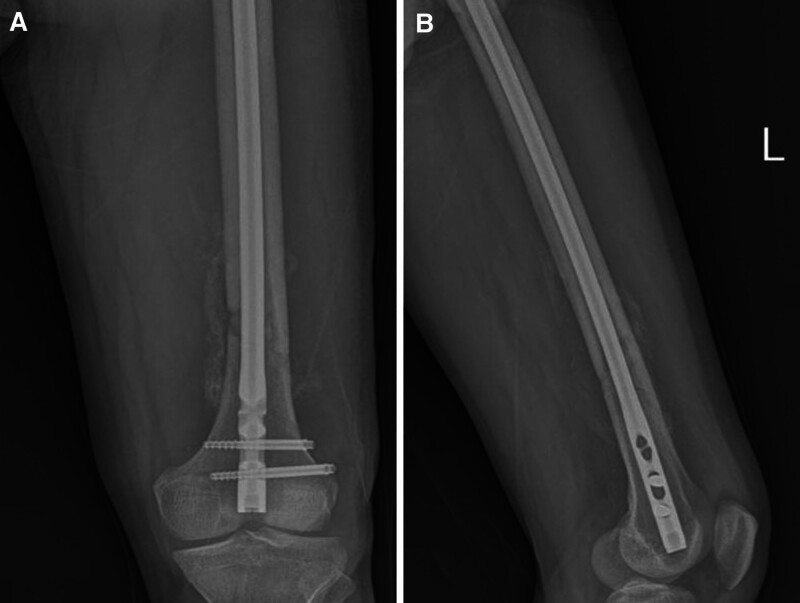
Anteroposterior (A) and lateral (B) radiological views at 8 months postoperatively of a 27-year-old patient who underwent retrograde femoral nailing with the diagnosis of femoral diaphyseal fracture (AO type 32A2). AO = Arbeitsgemeinschaft für Osteosynthesefragen.

The patient was positioned supine on a radiolucent table, and the distal femur was supported with a radiolucent rolled pad to maintain the knee joint at 20° to 30° flexion. A 2 to 3 cm longitudinal medial parapatellar incision was preferred to prepare the nail entry point. A guidewire was positioned above the posterior cruciate ligament attachment point at the intercondylar notch under fluoroscopic guidance and inserted intramedullary along the femoral diaphysis. The nail entry hole and medulla were reamed over the guidewire using a reamer of appropriate diameter. The appropriate nail length was measured, and the nail was placed intramedullary over the K-wire guide. The K-wire was removed and locking screws were placed in the distal and proximal holes using an external guide and fluoroscopy (Fig. 2).

#### 2.1.2. Suprapatellar approach tibia nailing

**Figure 3.**
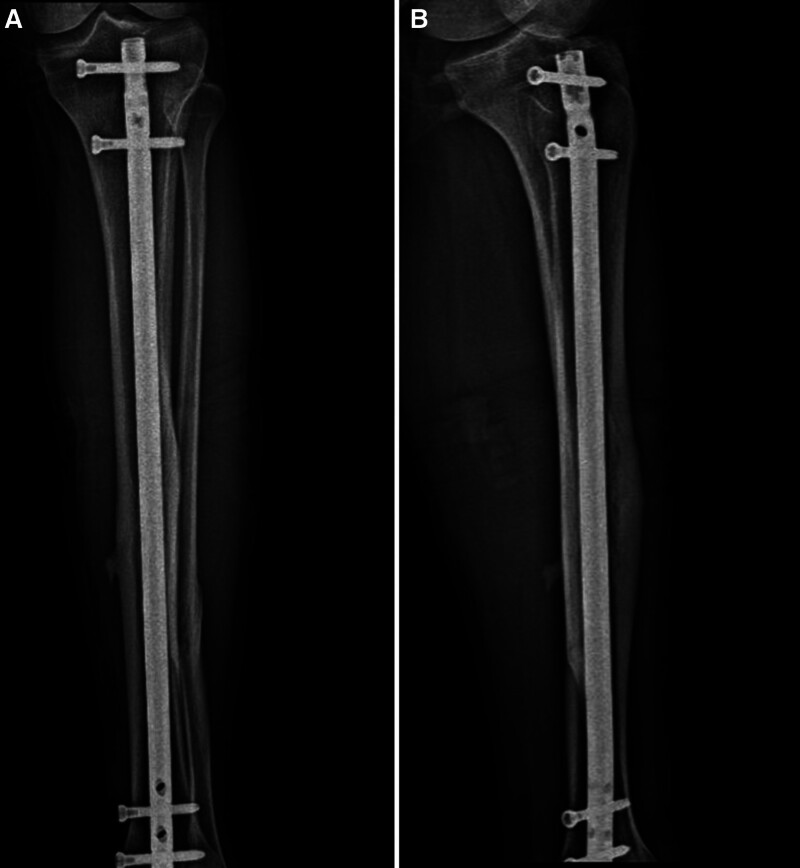
Final follow up radiological anteroposterior (A) and lateral (B) views at 30 months postoperatively of a 24-year-old male patient who underwent suprapatellar approach tibial nailing with the diagnosis of tibial diaphyseal fracture (AO type 42A1). AO = Arbeitsgemeinschaft für Osteosynthesefragen.

The patient was placed supine on a radiolucent table. A radiolucent rolled pad was placed under the knee joint to maintain 20° to 30° of flexion. The patella was marked and a longitudinal skin incision of approximately 2 cm was made approximately 1 cm proximal to the superior pole of the patella. Blunt dissection exposed the quadriceps tendon, which was split longitudinally along the midline to access the joint. The joint tightness was checked manually. If necessary, an additional incision was made in the upper two-thirds of the lateral or medial retinaculum. A guide K-wire was advanced through the joint into the proximal tibia. The ideal entry point, confirmed by fluoroscopy, is 8 to 10 mm lateral to the center of the tibial plateau in the anteroposterior view and at the oblique edge, extending distally from the anterior joint surface in the lateral view. At this stage, a multihole sleeve was used to identify the optimal entry point. Once the K-wire passed through the entry point, it was directed intramedullary. The subsequent preparation of the entry hole and medulla, nail insertion, and fixation with locking screws were performed in a manner similar to the RGF nailing technique (Fig. 3).

### 2.2. Statistical analysis

All analyses were performed using the SPSS software version 25.0 for Windows (IBM Corp. New York). Nonparametric statistical tests were used based on the presence of non-normally distributed data, as determined by the Shapiro–Wilk test. Descriptive statistics are expressed as mean, standard deviation, median, 1st to 3rd quartile, minimum and maximum values for numerical variables, and numbers and percentages for categorical variables. The Wilcoxon signed-rank test was used to compare ordinal data before and after the treatment. The intraclass correlation coefficient (ICC) was used to assess inter-observer agreement for the radiological KLOA classification system. Two-way random mode was used to calculate the ICC, with a 95% confidence interval. The ICC was interpreted as follows: < 0.50, poor; 0.50 and 0.75, fair; 0.75 and 0.90, good; and > 0.90, excellent. Statistical significance was set at *P* value < .05 was considered statistically significant.

## 3. Results

The study included 35 patients with a mean age of 39.0 ± 13.7 years (range: 19–65 years), comprising 32 males (91.4%) and 3 females (8.6%). The most common type of trauma among all patients was motor vehicle accidents (42.9%). No comorbidities were detected in 97.1% of patients. The most frequent accompanying injury was a multifracture (22.9%), while no additional injuries were identified in 65.7% of the patients. The median follow-up period was 11 months and the median fracture healing time was 5 months. At follow-up, 30 patients (85.7%) healed without complications, whereas stiffness was observed in 1 patient (2.9%) and delayed union in 4 patients (11.4%) (Table 1).

**Table 1:** Demographic characteristics of patients.

Parameters		Total (n:35)	Group 1 (n:21)	Group 2 (n:14)
Age		39.0 ± 13.7	42.3 ± 14.7	34.0 ± 10.7
Sex	Male	32 (91.4)	18 (85.7)	14 (100.0)
	Female	3 (8.6)	3 (14.3)	0 (0.0)
Side	Right	13 (37.1)	10 (47.6)	3 (21.4)
	Left	22 (62.9)	11 (52.4)	11 (78.6)
Fracture type (AO Classification)	32A1		6 (28.6)	–
	32A2		7 (33.3)	–
	32B2		4 (19.1)	–
	32B3		2 (9.5)	–
	32C3		2 (9.5)	–
	42A1		–	3 (21.4)
	42A2		–	9 (64.3)
	42B3		–	2 (14.3)
Type of trauma	Falling	11 (31.4)	8 (38.1)	3 (21.4)
	Vehicle accident	15 (42.9)	5 (23.8)	10 (71.4)
	Gunshot wound	7 (20.0)	6 (28.6)	1 (7.2)
	Pathological fracture	2 (5.7)	2 (9.5)	0 (0.0)
Comorbidities	None	34 (97.1)	20 (95.2)	14 (100.0)
	Hypertension	3 (8.6)	3 (14.3)	0 (0.0)
	Diabetes mellitus	1 (2.9)	1 (4.8)	0 (0.0)
	Malignancy	2 (5.7)	2 (9.5)	0 (0.0)
	Benign prostatic hyperplasia	1 (2.9)	1 (4.8)	0 (0.0)
Accompanying injuries	None	23 (65.7)	13 (61.9)	10 (71.4)
	Head trauma	1 (2.9)	0 (0.0)	1 (7.1)
	Multiple fracture	8 (22.9)	4 (19.0)	4 (28.6)
	Open fracture	3 (8.6)	3 (14.3)	0 (0.0)
	Neurological injury	1 (2.9)	1 (4.8)	0 (0.0)
	Shoulder dislocation	1 (2.9)	1 (4.8)	0 (0.0)
Complications	None	30 (85.7)	17 (81.0)	13 (92.9)
	Stiffness	1 (2.9)	1 (4.7)	0 (0.0)
	Delayed union	4 (11.4)	3 (14.3)	1 (7.1)
Fracture healing time		5 (4.0–6.0)	5.0 (4.0–6.0)	4.5 (3.75–5.75)
Follow-up period		11 (8.0–24.0)	10 (8.0–21.0)	11 (8.8–25.0)

Numerical data were summarized as mean ± standard deviation or median (1st–3rd quartile), and categorical data were summarized as numbers (percentage).

AO = Arbeitsgemeinschaft für Osteosynthesefragen.

RGF nailing (group 1) was performed in 21 patients (60.0%) and SPT nailing (group 2) in 14 patients (40.0%). In Group 1, 85.7% of the patients were male, whereas all patients in Group 2 were male. The most common types of trauma in group 1 were falls (38.1%) and gunshot injuries (28.6%), whereas in group 2, motor vehicle accidents (71.4%) and falls (21.4%) were the leading causes. The most common fracture type in Group 1 was 32A2 (33.3%) and in Group 2 was 42A2 (64.3%). The median fracture healing time was 5 months in group 1 and 4.5 months in group 2. No complications were observed in 81.0% and 92.9% of the patients in Groups 1 and 2, respectively (Table 1).

The distribution of the patients according to the KLOA classification system is shown in Table 2. In Group 1, the preoperative TFJ grades were most commonly grade 0 (76.2%) and grade 1 (23.8%). Following postoperative follow-up, 61.9% of the patients were grade 0 and 38.1% were grade 1. Similarly, the preoperative PFJ grades in this group were predominantly grade 0 (71.4%) and grade 1 (28.6%). However, in the postoperative period, the PFJ distribution was 52.4% (grade 1) and 42.9% (grade 0). In Group 2, the preoperative TFJ KLOA classification showed that 64.3% of patients were grade 0 and 28.6% were grade 1. Postoperatively, 57.1% remained grade 0, and 28.6% were grade 1. For the PFJ, 57.1% of patients were grade 0 and 35.8% were grade 1 preoperatively. By the end of the postoperative follow-up, 42.9% and 42.9% of the patients were classified as grade 0 and grade 1, respectively. All radiological KLOA classifications demonstrated significant interobserver agreement (Table 3). According to these results, the change in PFJ KLOA classification distribution between the preoperative and postoperative assessments in Group 1 was statistically significant (*P* = .023). In contrast, the changes in the TFJ KLOA classification in Group 1 (*P* = .059) and in both the TFJ and PFJ KLOA classifications in Group 2 (*P* = .317 and *P* = .083, respectively) were not statistically significant (Table 2).

**Table 2:** Distribution and comparison of preoperative and postoperative Kellgren and Lawrence classification of PFJ and TFJ.

Parameters	Group 1	Group 2
	n: 21 (60.0)	n: 14 (40.0)
*Preoperative TFJ*		
0	16 (76.2)	9 (64.3)
1	5 (23.8)	4 (28.6)
2	0 (0.0)	0 (0.0)
3	0 (0.0)	0 (0.0)
4	0 (0.0)	1 (7.1)
*Postoperative TFJ*		
0	13 (61.9)	8 (57.1)
1	8 (38.1)	4 (28.6)
2	0 (0.0)	0 (0.0)
3	0 (0.0)	1 (7.1)
4	0 (0.0)	1 (7.1)
*P* *	.059	.317
*Preoperative PFJ*		
0	15 (71.4)	8 (57.1)
1	6 (28.6)	5 (35.8)
2	0 (0.0)	1 (7.1)
3	0 (0.0)	0 (0.0)
4	0 (0.0)	0 (0.0)
*Postoperative PFJ*		
0	9 (42.9)	6 (42.9)
1	11 (52.4)	6 (42.9)
2	1 (4.8)	1 (7.1)
3	0 (0.0)	1 (7.1)
4	0 (0.0)	0 (0.0)
*P* *	.023	.083

Data were summarized as numbers (percentage).

PFJ = patellofemoral joint, TFJ = tibiofemoral joint.

* Wilcoxon signed ranks test was used.

**Table 3:** Intraclass correlation coefficient for inter-observer the Kellgren and Lawrence classification grading.

Parameters	ICC	%95 Confidence interval	*P*
Preoperative TFJ	0.937	0.875–0.968	<.01
Postoperative TFJ	0.927	0.855–0.963	<.01
Preoperative PFJ	0.917	0.836–0.958	<.01
Postoperative PFJ	0.986	0.972–0.993	<.01

ICC = Intraclass correlation coefficient, PFJ = patellofemoral joint, TFJ = Tibiofemoral joint.

## 4. Discussion

The main finding of our study, which assessed the effect of intramedullary nail systems performed through the knee joint on knee osteoarthritis, was that patients treated with RGF nail fixation showed a significant increase in PFJ KLOA grading in the early to-midterm follow-up compared to the preoperative period. In contrast, RGF nail fixation did not appear to affect the development of osteoarthritis in the TFJ. Additionally, SPT nail fixation had no effect on osteoarthritis in either the TFJ or the PFJ. In addition, intramedullary nail fixation, which was performed while preserving the biological environment necessary for fracture healing, resulted in low complication rates and rapid bone union.

### 4.1. Results and union time in RGF and SPT nails

Intramedullary nail fixation is widely preferred for the treatment of long-bone fractures of the lower extremities. In treating distal femoral fractures, a comparison of locking plate and RGF nail fixation indicates that RGF nail fixation results in faster bone healing and lower infection rates. However, no significant differences were observed between the 2 methods in other clinical indicators such as nonunion, delayed union, intraoperative blood loss, operation time, complication rates, reoperation rates, and functional outcomes.^[11]^ It has been reported that union is achieved in an average of 6 months with RGF nails, with 11.8% of cases resulting in nonunion.^[12]^ In a study comparing different implant types (RGF nail, antegrade femur nail, and distal femoral locking plate) for treating distal femur fractures, all 3 implants demonstrated similar bone-healing times. The same study noted that nails were associated with lower nonunion rates and better functional outcomes.^[13]^ Recently, the use of SPT nails has become increasingly common, particularly in proximal diaphyseal fractures of the tibia. This method offers several advantages, including improved fracture reduction, reduced risk of unexpected migration of the proximal fragment during fixation, decreased fluoroscopy time, better functional outcomes than IP nails, and less postoperative pain, which is commonly seen with IP tibial nails.^[14–16]^ A recent high-volume study reported an average union time of 27.2 weeks with SPT nails, achieving fracture healing in 97.6% of patients.^[17]^ In our study, bone healing was observed at an average of 5 months, with a delayed union rate of 14.3% in patients treated with RGF nails. On the other hand, union was achieved in 4.5 months without any complications in 92.9% of patients treated with SPT nails.

### 4.2. Iatrogenic cartilage damage and effects of nail fixation systems on osteoarthritis

A meticulous approach is essential to avoid iatrogenic cartilage damage in orthopedic intraarticular surgical procedures. This has been well-documented in many studies, especially in arthroscopic surgeries.^[18–20]^ However, in the available and current literature, studies emphasizing the effect of intramedullary nail implants (RGF and SPT nails) performed through the knee joint on cartilage damage or joint osteoarthritis are limited. Most existing studies have focused on knee pain observed after fixation. In a study conducted with RGF nails in sheep, it was reported that macroscopic and radiological findings of PFJ osteoarthritis started at the 3rd postoperative month and showed a significant increase at the 9th month. In the same study, degenerative changes in the TFJ were nearly absent.^[21]^ In another comparative study, 20% of the patients who underwent RGF nail fixation experienced postoperative knee pain. However, a direct correlation between knee pain and cartilage damage or osteoarthritis has not yet been established.^[22]^ In a case report of 2 patients, postoperative knee pain was attributed to iatrogenic cartilage damage in the trochlear groove (PFJ) due to malposition of the intramedullary nail entry point.^[8]^ In a biomechanical study performed on cadavers, positioning the nail 1 mm outside the cartilage level resulted in a significant increase in the contact pressure in the PFJ.^[23]^ Moreover, improper coronal and sagittal plane angulations during fracture reduction with femoral intramedullary nailing may lead to asymmetrical axial loading.^[24]^ The findings of the current study revealed a significant increase in the PFJ osteoarthritis KLOA grading in patients who underwent RGF intramedullary nails. No significant progression was observed in TFJ osteoarthritis. This finding does not support our hypothesis regarding RGF nails. However, we believe that these results should be corroborated by prospective studies with a larger number of patients and long-term follow-ups to establish their validity.

Although the SPT nail is associated with less knee pain and higher patient satisfaction than the IP nail, the risk of cartilage damage remains a concern. In fact, a cadaver study demonstrated cartilage damage in one-third of the cases when using the SPT nail.^[25]^ In a prospective study in which pre- and post-nail insertion arthroscopy was performed on patients with SPT nails, it was reported that chondromalacia detected on magnetic resonance imaging performed in the 1st year was not correlated with either pre- or post-nail insertion arthroscopy; furthermore, it was reported that patients with PFJ changes observed on post-nail insertion arthroscopy did not experience knee pain during the 1st year.^[26]^ In contrast, Gelbke et al found that high PFJ contact pressures during SPT nailing were below the threshold required to cause cartilage damage.^[27]^ In our study, which evaluated the relationship between postoperative osteoarthritis in the PFJ and TFJ, no significant progression was observed in KLOA grading in patients who underwent SPT tibial nailing. This finding supports our research hypothesis regarding SPT tibial nails.

## 5. Limitations and prospects

The limitations of our study were its retrospective nature, small sample size of the groups, early-medium follow-up period, lack of radiological (such as magnetic resonance) or arthroscopic findings regarding preoperative and early postoperative cartilage status, and evaluation of only radiological changes in terms of osteoarthritis. Another limitation of this study is the lack of a control group (e.g., healthy contralateral knees) to compare the results, as only preoperative and postoperative radiographs of the injured side were available. However, we believe that our study focusing on the effects of intramedullary nail systems performed through the knee joint on the development of osteoarthritis will be helpful for future comprehensive research.

## 6. Conclusion

RGF and SPT nails demonstrated a rapid fracture healing time and low complication rate. The SPT nail did not affect the early KLOA grade in terms of osteoarthritis risk for both the PFJ and TFJ of the knee joint. Although the RGF nail had no effect on osteoarthritis in the TFJ, it was found to show progression in the KLOA grade in the PFJ. More comprehensive studies with larger numbers of patients are needed to validate our results.

## Author contributions

**Conceptualization:** Ahmet Senel, Baris Acar.

**Data curation:** Baris Acar, Ahmet Sinan Kalyenci.

**Formal analysis:** Ahmet Senel, Baris Polat.

**Software:** Engin Carkci, Sinan Erdogan.

**Writing – original draft:** Ahmet Senel.

**Writing – review & editing:** Engin Carkci, Baris Polat, Erhan Sukur.

## References

[1] Bode G Strohm PC Südkamp NP Hammer TO. Tibial shaft fractures - management and treatment options. A review of the current literature. Acta Chir Orthop Traumatol Cech. 2012;79:499–505. 23286681

[2] Nino S Courington R Brooks P Langford J Haidukewych G. Retrograde nailing for extremely proximal fractures of the femoral shaft. J Orthop Trauma. 2023;37:346–50. 36821474 10.1097/BOT.0000000000002586

[3] Cui Y Hua X Schmidutz F Zhou J Yin Z Yan SG. Suprapatellar versus infrapatellar approaches in the treatment of tibia intramedullary nailing: a retrospective cohort study. BMC Musculoskelet Disord. 2019;20:573. 31779596 10.1186/s12891-019-2961-x PMC6883512

[4] Rodríguez-Zamorano P García-Coiradas J Galán-Olleros M. Suprapatellar tibial nailing, why have we changed? Rev Esp Cir Ortop Traumatol. 2022;66:159–69. 35590432 10.1016/j.recot.2021.09.008

[5] Jayaraju U Rammohan R Awad F. Tibial intramedullary nailing by suprapatellar approach: is it quicker and safer? Cureus. 2022;14:e29915. 36348901 10.7759/cureus.29915 PMC9633433

[6] Lane JG Smith J. Trochlear lesion caused by suprapatellar intramedullary nailing and treated with autologous chondrocytes implant. J Orthop Case Rep. 2022;12:1–5. 10.13107/jocr.2022.v12.i03.2690 PMC9499059 36199915

[7] Breyer G Usmani K Hwang R Begley B Mashru RP Gutowski CJ. Knee pain and functional outcomes after retrograde femoral nailing: a retrospective review. Arch Bone Jt Surg. 2023;11:218–24. 37168582 10.22038/ABJS.2022.67164.3215 PMC10165212

[8] DePhillipo NN Lebus GF Cinque ME Kennedy NI Chahla J LaPrade RF. Iatrogenic trochlear chondral defects after anterolateral placement of retrograde femoral nails. Arthroscopy. 2017;33:1727–30. 28754245 10.1016/j.arthro.2017.06.004

[9] Meinberg EG Agel J Roberts CS Karam MD Kellam JF. Fracture and dislocation classification compendium-2018. J Orthop Trauma. 2018;32(Suppl 1):S1–S170. 10.1097/BOT.0000000000001063 29256945

[10] Kellgren JH Lawrence JS. Radiological assessment of osteo-arthrosis. Ann Rheum Dis. 1957;16:494–502. 13498604 10.1136/ard.16.4.494 PMC1006995

[11] Kim HS Yoon YC Lee SJ Sim JA. Which fixation produces the best outcome for distal femoral fractures? Meta-analysis and systematic review of retrograde nailing versus distal femoral plating in 2432 patients and 33 studies. Eur J Trauma Emerg Surg. 2024;50:763–80. 38057606 10.1007/s00068-023-02393-8

[12] Chandra Vemulapalli K Pechero GR Warner SJ. Is retrograde nailing superior to lateral locked plating for complete articular distal femur fractures? Injury. 2022;53:640–4. 34863509 10.1016/j.injury.2021.11.037

[13] Gönder N Özel V Kaya O Demir IH. Comparison of retrograde intramedullary nailing, antegrade intramedullary nailing and distal femur locked plating methods in the treatment of extra-articular distal femur fractures: a retrospective analysis: Treatment of extraarticular distal femur fractures. The Injector. 2023;2:253–60.

[14] Çiçekli O Kochai A Şükür E Başak AM Kurtoğlu A Türker M. Suprapatellar approach for fractures of the tibia: does the fracture level matter? Eklem Hastalik Cerrahisi. 2019;30:10–6. 30885103 10.5606/ehc.2019.63487

[15] Gao Z Han W Jia H. Suprapatellar versus infrapatellar intramedullary nailing for tibal shaft fractures: a meta-analysis of randomized controlled trials. Medicine (Baltim). 2018;97:e10917. 10.1097/MD.0000000000010917 PMC6023710 29901581

[16] Lu K Wu ZQ Qian RX Gao YJ. The efficacy of suprapatellar, parapatellar and infrapatellar intramedullary nailing in the treatment of tibial fractures: a systematic review and meta-analysis. Arch Orthop Trauma Surg. 2024;144:4917–27. 39325161 10.1007/s00402-024-05584-z

[17] Teixidor-Serra J Andrés-Peiró JV García-Sanchez Y. Outcomes and their predictors in suprapatellar nailing for tibia fractures. Multivariable analysis of 293 consecutive cases. Eur J Trauma Emerg Surg. 2024;50:1577–84. 38472386 10.1007/s00068-024-02476-0

[18] Claret G Montañana J Rios J. The effect of percutaneous release of the medial collateral ligament in arthroscopic medial meniscectomy on functional outcome. Knee. 2016;23:251–5. 26652573 10.1016/j.knee.2015.07.013

[19] Bosco F Giustra F Ghirri A Battaglia DL Capella M Massè A. The pie-crust surgical technique for medial collateral ligament release: enhancing visualization of the medial compartment in knee arthroscopy. Ann Jt. 2024;9:14. 38694812 10.21037/aoj-23-54 PMC11061655

[20] Fakioglu O Ozsoy MH Ozdemir HM Yigit H Cavusoglu AT Lobenhoffer P. Percutaneous medial collateral ligament release in arthroscopic medial meniscectomy in tight knees. Knee Surg Sports Traumatol Arthrosc. 2013;21:1540–5. 22766688 10.1007/s00167-012-2128-x

[21] Pingsmann A Lederer M Wüllenweber C Lichtinger TK. Early patellofemoral osteoarthritis caused by an osteochondral defect after retrograde solid nailing of the femur in sheep. J Trauma. 2005;58:1024–8. 15920419 10.1097/01.ta.0000171986.10452.f4

[22] Gill S Mittal A Raj M Singh P Singh J Kumar S. Extra articular supracondylar femur fractures managed with locked distal femoral plate or supracondylar nailing: a comparative outcome study. J Clin Diagn Res. 2017;11:RC19–23. 28658862 10.7860/JCDR/2017/25062.9936 PMC5483764

[23] Morgan E Ostrum RF DiCicco J McElroy J Poka A. Effects of retrograde femoral intramedullary nailing on the patellofemoral articulation. J Orthop Trauma. 1999;13:13–6. 9892119 10.1097/00005131-199901000-00004

[24] Aliç T Güler C Çalbiyik M Hassa E. Which of the three different intramedullary nail designs is superior in the treatment of femoral shaft fractures? J Health Sci Med. 2023;6:467–75.

[25] Zamora R Wright C Short A Seligson D. Comparison between suprapatellar and parapatellar approaches for intramedullary nailing of the tibia. Cadaveric study. Injury. 2016;47:2087–90. 27461777 10.1016/j.injury.2016.07.024

[26] Chan DS Serrano-Riera R Griffing R. Suprapatellar versus infrapatellar tibial nail insertion: a prospective randomized control pilot study. J Orthop Trauma. 2016;30:130–4. 26894640 10.1097/BOT.0000000000000499

[27] Gelbke MK Coombs D Powell S DiPasquale TG. Suprapatellar versus infra-patellar intramedullary nail insertion of the tibia: a cadaveric model for comparison of patellofemoral contact pressures and forces. J Orthop Trauma. 2010;24:665–71. 20926959 10.1097/BOT.0b013e3181f6c001

